# Early Diagnosis of Delirium in Geriatric Patients in Emergency Departments: A Systematic Review and Meta-Analysis

**DOI:** 10.3390/brainsci16080864

**Published:** 2026-08-15

**Authors:** Paula Albusac-Olivares, Sara Chami-Peña, José M. Gutiérrez-Pastor, Alberto Caballero-Vázquez, Miguel Quesada-Caballero, Nora Suleiman-Martos, Guillermo A. Cañadas-De la Fuente, José Luis Romero-Béjar, María José Membrive-Jiménez

**Affiliations:** 1 Sierra de Cazorla High-Resolution Hospital, Andalusian Health Service, Carretera A-319 Cazorla-Peal del Becerro, s/n, 23470 Jaén, Spain; paulaalbusacolivares@gmail.com; 2 Centro de Salud de Santa Fe, Distrito Sanitario Granada-Metropolitano, Servicio Andaluz de Salud, Calle Virgen de la Consolación, 12, 18015 Granada, Spain; 3 San Cecilio Clinical University Hospital, Andalusian Health Service, Av. del Conocimiento s/n, 18016 Granada, Spain; gutiforecast37@gmail.com; 4 DiagnosticLung Cancer Unit, Broncopleural Techniques and Interventional Pulmonology Departament, Hospital Universitario Virgen de las Nieves, 18014 Granada, Spain; alberto.caballero.sspa@juntadeandalucia.es; 5 Institute for Biosanitary Research (ibs.GRANADA), 18012 Granada, Spain; miguel.quesada.caballero.sspa@juntadeandalucia.es (M.Q.-C.); jlrbejar@ugr.es (J.L.R.-B.); mjmembrive@ugr.es (M.J.M.-J.); 6 Centro de Salud Albayda La Cruz, Distrito Sanitario Granada-Metropolitano, Servicio Andaluz de Salud, Calle Virgen de la Consolación, 12, 18015 Granada, Spain; 7 Faculty of Health Sciences, University of Granada, Av. Ilustración 60, 18016 Granada, Spain; norasm@ugr.es; 8 Brain, Mind and Behaviour Research Center (CIMCYC), University of Granada, Campus Universitario de Cartuja s/n, 18011 Granada, Spain; 9 Department of Statistics and Operations Research, University of Granada, Av. de Fuente Nueva, s/n, 18071 Granada, Spain; 10 Ceuta Faculty of Health Sciences, University of Granada, Cortadura del Valle s/n, 51001 Ceuta, Spain

**Keywords:** delirium, acute confusional state, emergency department, geriatrics, early diagnosis

## Abstract

Background: Acute confusional state, or delirium, is a common neuropsychiatric disorder in older adults, particularly in emergency departments. It is associated with high morbidity and mortality, as well as difficult detection. Its early identification is crucial to prevent complications and improve prognosis. This study aims to identify and analyze available strategies for the early diagnosis of delirium in geriatric patients in emergency departments. Methods: A literature review and meta-analysis were conducted between 2020 and 2026 using the PubMed, Scopus, Web of Science, CINAHL, and Cochrane databases (PRISMA 2020). To estimate the prevalence of patients with delirium in emergency departments, a meta-analysis was performed using Stats Direct statistical software (Version 4). Methodological quality was evaluated according to the Oxford Centre for Evidence-Based Medicine levels of evidence. Results: Eleven studies were included, demonstrating that delirium in emergency departments is highly prevalent among older adults, particularly those with dementia, polypharmacy, or functional impairment. The 4AT scale was the most frequently used screening tool, notable for its strong sensitivity. Hypoactive delirium emerged as the most common subtype and carried the poorest prognosis. Furthermore, its presence was associated with longer hospital stays, increased complications, and higher mortality rates, while a lack of training among healthcare staff continues to limit early detection. The meta-analysis detected no publication bias and revealed a 20.6% prevalence of delirium among patients in emergency departments. Conclusions: One in five patients attending the emergency department presents with delirium. Delirium in geriatric emergency care demands a structured clinical response grounded in prevention, early detection, and professional training. Integrating validated diagnostic tools and reinforcing the role of nursing professionals are key to improving patient outcomes and safety.

## 1. Introduction

Acute confusional state, or delirium, is a multifactorial neuropsychiatric disorder typical of critical illness, characterized by a sudden and fluctuating onset with marked nocturnal worsening. It leads to a decline in attention, transient alterations in consciousness, and impaired cognitive function, resulting in significant disability for affected individuals [1].

The various clinical presentations of delirium are classified based on the predominant alteration in psychomotor activity, distinguishing four main subtypes. First, hyperactive delirium is dominated by symptoms of agitation and a state of hypervigilance, and is generally associated with a more favorable prognosis. Conversely, hypoactive delirium is characterized by decreased activity, lethargy, and irritability, which often delays diagnosis and leads to a poorer prognosis. Mixed delirium involves alternating symptoms of the two aforementioned subtypes and can be reversible if the underlying triggers are treated. Finally, subsyndromal delirium presents with partial symptoms that do not fulfill all diagnostic criteria [2].

The aetiology is multifactorial and involves an interaction between the patient’s vulnerability and precipitating factors, which we know little about [3]. Therefore, special attention must be given to age, sex, educational level, high medication and drug consumption, medical comorbidities (such as infections or inadequate pain management), sensory impairment, malnutrition with vitamin deficiencies, and functional and psychiatric status. Furthermore, there is an iatrogenic risk associated with prolonged stays in emergency departments (EDs) and the use of physical restraints [4].

Various therapeutic strategies currently exist, with non-pharmacological approaches being prioritized. These strategies are effective in reducing the incidence of delirium and improving the prognosis in older patients [5]. These include spatio-temporal reorientation using calendars or clocks, adequate hydration, sleep improvement, and family accompaniment. This is favored over pharmacological therapy (e.g., antipsychotics or benzodiazepines)—which lacks high-quality scientific evidence demonstrating significant improvement—reserving the latter only for severe agitation or withdrawal syndromes [6].

Although it can occur at any stage of life, the prevalence of this disorder is higher in individuals over 65 years of age (1–2%) in developed countries, increasing considerably in those over 85 years (10–14%), and is more pronounced in males, hospitalized patients, or those with compromised health. In EDs, delirium affects 10–13% of older adults and is associated with a threefold increase in mortality, reaching a rate similar to that of myocardial infarction or sepsis [7]. Moreover, delirium can accelerate the effects of Alzheimer’s disease and contribute to greater long-term cognitive decline, even in individuals with a normal baseline cognitive status [8]. The frequency of delirium varies across populations, clinical settings, and assessment methods; however, this is not the only factor, as underdiagnosis and the variability of detection methods used by healthcare professionals can significantly skew the data. This is the case in developing countries, where prevalence rates reach as high as 55.4% and 35–50% of cases remain undiagnosed [9].

Diagnosing this disorder becomes an arduous task for healthcare professionals due to the wide disparity of detection methods, limited knowledge for conducting a differential diagnosis given its heterogeneous symptomatology, and the poor implementation of available tools [10].

The early detection of delirium is fundamental, as it is a common and severe condition in geriatric patients attending EDs. Early identification improves access to recommended treatment pathways, management of associated risks, provision of prognostic information, and reduction in hospital stays. It also facilitates high-quality diagnostic communication with patients and caregivers, ultimately aiming to reduce incidence, prevent complications, and alleviate disease burden and healthcare costs [11]. Therefore, the aim of this review was to identify the best method for diagnosing delirium in geriatric patients in EDs.

## 2. Materials and Methods

### 2.1. Search Strategy

A review of the scientific literature and a meta-analysis were conducted using the PubMed, Scopus, Web of Science, CINAHL, and Cochrane databases, in accordance with the provisions of the PRISMA 2020 statement (Table S1). The research was registered in the PROSPERO database (ID: CRD420261445892).

The following search equation was employed: “(delirium OR acute confusional state) AND (geriatrics OR elderly OR older adults) AND (emergency department OR emergency services) AND (early diagnosis OR diagnostic tools OR early detection OR screening)”, as well as its Spanish equivalent. The search terms within the equation were selected from the Medical Subject Headings (MeSH) thesaurus. The search ended on 31 January 2026.

### 2.2. Eligibility Criteria

Inclusion and exclusion criteria were systematically applied during the study selection process:

Inclusion criteria: Full-text quantitative primary sources addressing diagnostic strategies for delirium in geriatric patients within ED settings, published in either English or Spanish between 2020 and 2026.

Exclusion criteria: Doctoral dissertations, articles unrelated to the study objective or target population, and studies involving mixed samples that did not report outcomes separately.

### 2.3. Variables and Data Collection

A dedicated data collection sheet was developed, comprising the following variables: authorship, year of publication, methodological design, prevalence, primary outcomes, and level of evidence (Table A1).

### 2.4. Study Selection Process

Two independent reviewers participated in the selection process. Initially, articles were screened by title and abstract, followed by a comprehensive full-text reading. Finally, a reverse search was performed by cross-referencing the bibliographies of the selected articles.

### 2.5. Critical Appraisal and Level of Evidence

The methodological quality of the included studies was appraised in accordance with the levels of evidence and grades of recommendation established by the Oxford Centre for Evidence-Based Medicine (OCEBM) [12].

### 2.6. Quality Assessment and Bias Evaluation

StatsDirect statistical software (Version 4) was utilized to perform a random-effects meta-analysis to estimate the prevalence of ED patients presenting with delirium. To calculate this prevalence, the total sample size and the number of cases diagnosed with delirium in the ED were extracted. Heterogeneity was evaluated using the $I^{2}$ statistic, and publication bias was assessed using Egger’s test.

The methodological quality and internal risk of bias of the included studies were formally assessed using the Joanna Briggs Institute (JBI) Critical Appraisal Checklist for Studies Reporting Prevalence Data [13]. Each study was evaluated across 9 specific domains: sample frame appropriateness, sampling strategy, sample size adequacy, detailed description of study subjects and setting, coverage of data analysis, use of valid diagnostic tools, standardized measurement, appropriate statistical analysis, and response rate handling (Table S2).

## 3. Results

**Figure A1:**
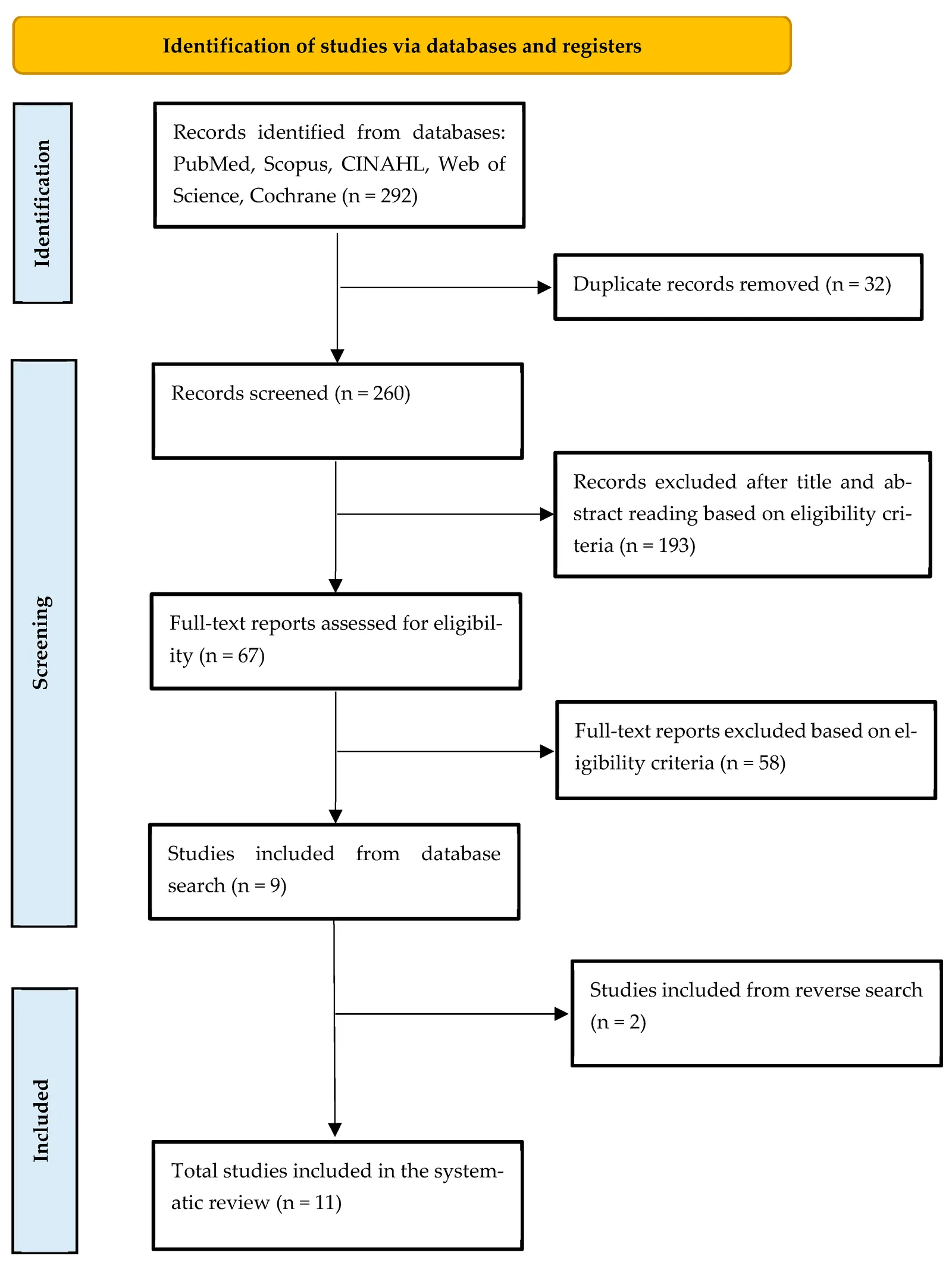
Flowchart of the study selection process.

The initial search yielded 1682 articles; after applying the exclusion criteria, this was reduced to 292, of which 32 were duplicates. Of the remaining 260 documents, 193 were discarded after reading the title and abstract. Following a critical full-text reading of the articles, 9 were obtained. Finally, a reverse search was performed, resulting in a final sample of n = 11 articles. The flowchart of the selection process is shown in Figure A1. The main characteristic of the studies are shown in the Table A1.

### 3.1. Sociodemographic and Clinical Characteristics of the Patients

Delirium has a higher prevalence in older adults, especially in the 76–84 age range. Most of the analyzed studies present a predominantly male sample [14,15,16,17,18,19,20,21,22], with the exception of the studies by Anand et al. [23] and Oliveira et al. [24], in which the majority of participants were women. However, no significant differences were found regarding sex distribution.

According to Soler-Sanchis et al. [20], patients with delirium present a higher daily consumption of prescription medications compared to those without a diagnosis. Furthermore, a higher frequency of altered Glasgow Coma Scale scores was observed within this group. Meged-Book et al. [16] pointed out a statistically significant difference in the presentation of delirium between single and married patients aged over 65, being higher in the former group (59.4%).

Regarding clinical status, Béland et al. [14] highlighted in their work that the delirious group had a lower functional status, a higher level of frailty, and was more cognitively impaired at baseline than the non-delirious group, a theory supported by the work of Anand et al. [23]. Moreover, a previous episode of confusion or delirium was more common among patients with delirium (47% vs. 6%) [16].

Finally, concerning the typology of delirium, although most articles do not specify which is the most frequent, the study by Oliveira et al. [24] identified hypoactive delirium as the predominant subtype in their analysis.

### 3.2. Risk Factors Associated with Delirium in the ED

According to the scientific literature, dementia is one of the primary risk factors associated with delirium. Soler-Sanchis et al. [21] noted that patients with this condition are three times more likely to develop it. These findings align with those reported by Pouw et al. [19], who identified that 22.5% of patients in the acute geriatric ward had a history of dementia. Similarly, Meged-Book et al. [16] observed that 44.1% of the 68 patients diagnosed with dementia presented with delirium, compared to 9.9% of those without this diagnosis. Lastly, Mueller et al. [18] supported this theory with 30.6% versus 4% in healthy patients.

There are other risk factors related to delirium, such as the presence of a previous stroke, acute neurological diseases, falls within the last 30 days, and polypharmacy [21].

Among the strongest predictors highlighted were the triage nurse’s chief complaint of an altered mental state and the presence of confusion identified during a fall risk assessment by a nurse [18]. Furthermore, other studies identified cognitive impairment, the lack of mobilization during the ED stay, and a prolonged length of stay in this department as main predictors [14]. Additionally, bizarre behavior, poor general condition, rapid onset, and recent neurological symptoms were also noted [21].

### 3.3. Screening Tools and Diagnostic Accuracy of Delirium in the ED

The 4A’s Test (4AT) is one of the most widely used tools for the early diagnosis of delirium in the ED. The cutoff point offering the highest diagnostic accuracy has been analyzed, observing that a score of 3 or more points on the scale provides the best sensitivity in the general sample [22].

For their part, Morales-Puerto et al. [17] carried out a Spanish adaptation of the 4AT scale, establishing the cutoff point at 4, with a sensitivity of 95.83% and a specificity of 92.98%. However, no significant differences were found in the detection of delirium when compared to the Delirium Rating Scale-Revised-98 (DRS-R-98).

Several studies have also evaluated other diagnostic tools. Meged-Book et al. [16] selected the brief Confusion Assessment Method (bCAM) and promoted awareness among the healthcare team regarding the importance of its detection, which increased the diagnosis rate to 37% during that period. Likewise, Mailhot et al. [15] opted for the Family Confusion Assessment Method (FAM-CAM) tool, which showed higher sensitivity in patients with dementia and superior overall specificity compared to the standard Confusion Assessment Method (CAM).

A more innovative approach was presented by Mueller et al. [18], who developed a machine learning model to identify patients at higher risk of delirium within the first three days following their admission to the ED. Among the evaluated models—Random Forest Classifier (RF), Gradient Boosting Classifier (GBM), and Logistic Regression (LR)—all three were found to have similar accuracy. However, the GBM model stood out for its higher specificity and sensitivity.

Finally, Oliveira et al. [24] utilized the REDEEM (REcognizing DElirium in Emergency Medicine) risk score, which evaluates 10 different variables. Their study revealed that a 10-unit increase in the risk score is associated with a threefold higher probability of developing delirium. Furthermore, they determined that a score equal to or greater than 11 on the Youden index indicates a high risk for the patient; however, to favor sensitivity, the optimal cutoff point was established at 5.

### 3.4. Impact of Screening on Care, Comorbidities, and Hospital Length of Stay

Hospital length of stay for patients with an abnormal score on the 4AT scale was more than double compared to patients without cognitive alterations [14,22]. However, readmission rates did not show significant differences in most studies, except in the city of Salford and in the study by Mailhot et al. [15].

The presence of delirium in patients attending the ED not only prolongs hospital stays and increases complications but is also associated with a statistically significant higher mortality rate compared to the group of patients without this condition. This relationship has been supported by multiple studies, including that of Morales-Puerto et al. [17].

Along these lines, the study by Anand et al. [23] demonstrated that the mortality rate was higher in patients who were not subjected to full screening with the 4AT scale. Moreover, within the evaluated group, the risk of death increased proportionally to the score obtained on the scale: 4% in patients with 4AT = 0; 7% in those with 4AT = 1–3; and up to 17% in those who scored 4AT = 4. Based on these data, it was concluded that the risk of 30-day hospital mortality was 5.5 times higher in patients with 4AT = 4 compared to those with 4AT = 0.

Similarly, Mailhot et al. [15] reported that the six-month mortality rate was significantly higher in patients with a positive result on the FAM-CAM scale (13% vs. 3%). Likewise, the Charlson Comorbidity Index (CCI), used to assess 10-year life expectancy, showed higher values in patients with delirium, suggesting a greater burden of comorbidities in this population [16].

### 3.5. Staff Training and Knowledge in Delirium Identification

The analyzed studies evidenced a lack of specific training in delirium detection among nursing staff. Vardy et al. [22] found that only 35% of healthcare professionals were aware of the existence of an accessible screening tool through the ED system. Furthermore, more than half of the respondents indicated that they did not use any structured method for delirium identification, reflecting a low implementation of these resources in clinical practice.

### 3.6. Meta-Analysis

**Figure A2:**
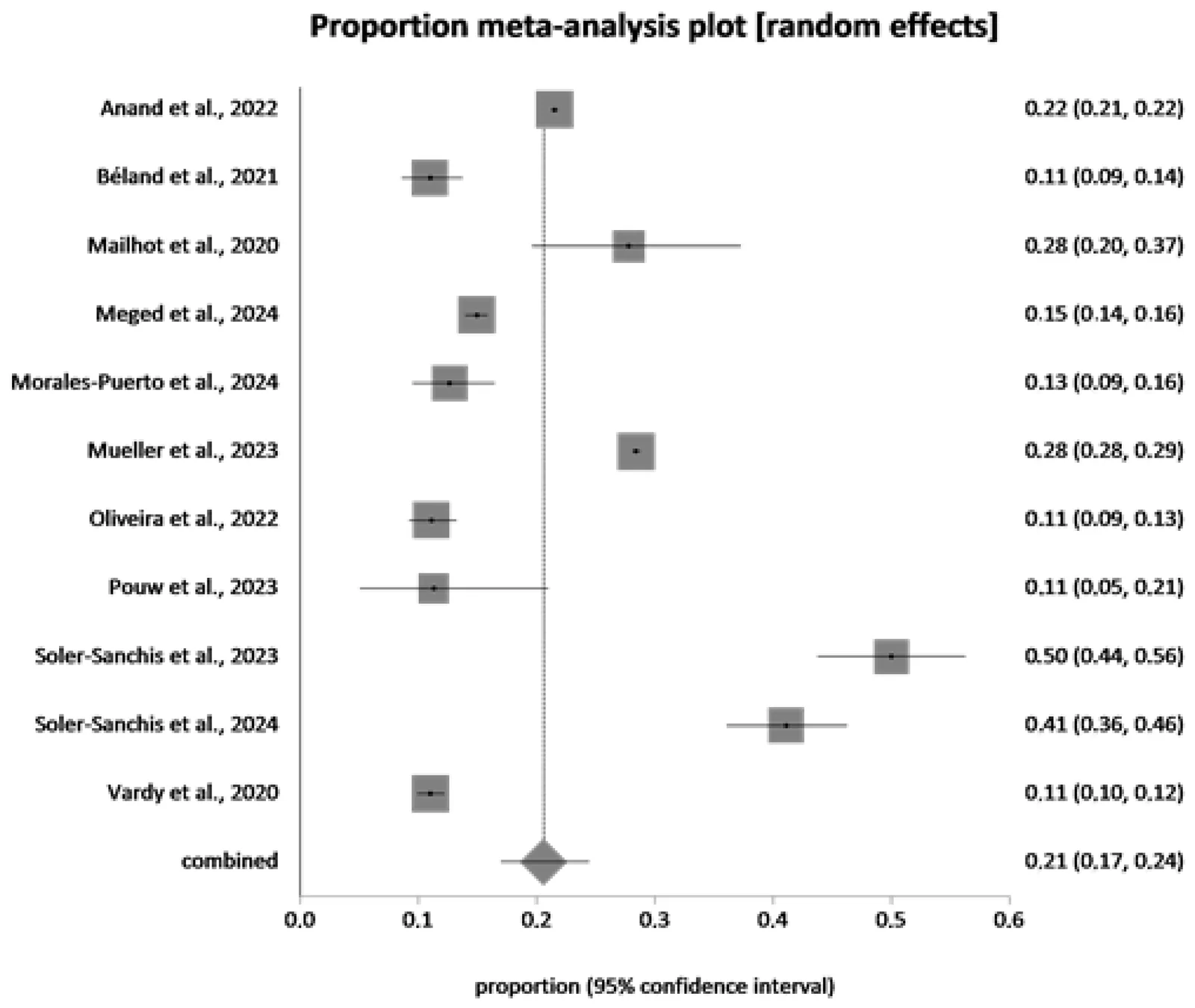
Forest plot of the prevalence of patients attending the emergency department with delirium [14,15,16,17,18,19,20,21,22,23,24].

A total of 11 studies were analyzed, and 164,478 patients were included in the meta-analysis. According to Egger’s test, no publication bias was detected in any of the cases. The $I^{2}$ value was 99.5%. The meta-analytical estimate of the percentage of patients attending the ED due to delirium was 20.6% (95% CI: 16.95–24.42%). The forest plot shown in Figure A2 displays the meta-analytical results.

### 3.7. Risk of Bias Assessment

Methodological quality assessment using the JBI Critical Appraisal Checklist showed that eight studies presented a low risk of bias [14,16,17,18,21,22,23,24], two studies exhibited a moderate risk of bias mainly attributable to smaller sample sizes [15,19], and one study presented a high risk of bias due to its retrospective design relying on routine clinical coding without active screening [20]. A detailed item-by-item breakdown of the risk of bias assessment is available in Supplementary Materials.

## 4. Discussion

Delirium in older patients constitutes a major clinical challenge in the ED setting due to its high prevalence and associated severe consequences. Its onset has been linked to multiple risk factors widely documented in the literature. According to the meta-analysis results, 20.6% of patients treated in the ED presented with a clinical picture of delirium, a prevalence that is consistent with the analyzed scientific literature [25]. Several studies have shown that aging significantly increases the likelihood of developing delirium [26,27]. Nonetheless, no significant differences have been identified regarding sex. Furthermore, it has been noted that patients presenting with delirium tend to exhibit a more deteriorated functional status compared to those who do not suffer from it. However, unlike the findings of the present study, those investigations did not identify significant variations based on marital status [28].

### 4.1. Risk Factors and Subtypes

In addition to age, other elements have shown a close relationship with the development of delirium. Among them, dementia stands out as the primary predisposing factor, a fact that can be explained by pathophysiological mechanisms such as cholinergic dysfunction, given that acetylcholine plays a fundamental role in attentional processes and consciousness [29,30]; neuroinflammation, which disrupts neural circuits and triggers the episode [31]; or oxidative stress, which causes long-term damage to neurons, contributing to dementia, and triggers acute episodes of delirium; amongst others [32].

Polypharmacy, especially the use of psychoactive drugs, constitutes another prominent risk factor, the use of which has been associated with an increased threat of developing delirium in older patients [33]. This may be due to adverse reactions produced by certain medications such as opioids, benzodiazepines, dihydropyridines, antidepressants, and antihistamines, which are frequently used in the hospital setting and primarily trigger hyperactive delirium. However, it is worth noting that in the geriatric population, the hypoactive delirium subtype is the most frequent, being the one that most often goes unnoticed, with a poorer prognosis and high mortality in emergency services [31]. Likewise, prolonged hospital stays have also been linked to a higher probability of developing delirium. Other factors contributing to increased risk include infections, dehydration, stroke, cardiovascular diseases, and the presence of severe pain, which have been highlighted as determinants in various studies [26,34].

### 4.2. Diagnostic and Screening Instruments

Regarding diagnostic assessment instruments, the 4AT scale has consolidated itself as the most widely used tool internationally for the rapid detection of delirium in the ED. Its high sensitivity (91.6%) and specificity (79.9% or 86.9–86.9%, respectively, according to studies such as that by Calf et al. [26]), along with its simplicity and speed of administration, explain its widespread adoption in emergency settings. Unlike other scales such as the Confusion Assessment Method (CAM), which requires between 10 and 20 min for application and specific training, the 4AT does not require prior training, making it a more viable option in clinical contexts with a high care burden [35,36,37,38].

The CAM tool, despite being widely validated (sensitivity of 86%, specificity of 93%), may present limitations due to its reliance on subjective clinical observation. In response to this limitation, the brief version (bCAM) was developed as a more objective, rapid, and easy-to-apply tool, even for professionals without prior experience. It features a sensitivity of 84% and a specificity of 96%, making it an effective alternative for the emergency environment [36]. Regarding the Family Confusion Assessment Method (FAM-CAM), the literature supports its moderate sensitivity (74%) and good specificity (91%), which is superior to the standard Confusion Assessment Method (CAM) [39].

Other studies have validated the efficacy of the Gradient Boosting Classifier (GBM) model, highlighting its high diagnostic performance. In particular, an area under the curve (AUC) of 0.933 was obtained, reflecting a superior predictive capacity compared to other models, as also observed in this review [36]. In the article by De Filippis et al. [38], the model showed an accuracy of 55%, demonstrating its utility in differentiating between stable and critical cases, thereby facilitating resource prioritization. However, the authors pointed out the need for further model refinement in complex clinical scenarios to reduce the false-negative rate.

Therefore, according to the scientific literature, of the tools analysed, the 4AT scale and the bCAM are considered the best for EDs. This is because they take less than three minutes to administer, do not require complex training, and are suited to environments with high patient volumes [39,40,41].

### 4.3. Clinical Impact, Mortality, and the Role of Nursing

Delirium screening has a significant clinical impact. Studies reveal that hospital complications were present in 13.48% of patients with delirium compared to 4.85%, and the hospital stay was longer in the former group than in the latter [42]. Among the most frequent associated comorbidities are nosocomial infections, urinary tract infections (UTIs), stroke, diabetes, hypertension, and pressure ulcers, among others [40]. In addition, acute confusional state was significantly associated with an increase in total healthcare costs [43].

Regarding mortality, according to Arneson et al. [44], patients discharged with delirium presented higher 30-day mortality and a higher probability of ED readmission compared to patients without this condition. Furthermore, it was evidenced that the combination of delirium or mild cognitive impairment along with a prolonged stay in the emergency room tripled the mortality risk. This risk increased up to sevenfold in cases where patients were subsequently hospitalized. This theory is supported by other studies showing that delirium remained significantly associated with an increase in 30-day mortality of 16.8% compared to 4.3% in those unaffected [45].

Finally, a lack of homogeneity in healthcare staff training regarding the use of these instruments has been identified. Kennedy et al. [45] warn that without adequate training on how to use delirium assessment instruments, the diagnostic accuracy of the tools is considerably reduced [46].

In this context, delirium prevention emerges as the most effective strategy to reduce its incidence and associated complications in older adults treated in EDs. This syndrome, especially in its hypoactive form, frequently goes unnoticed, highlighting the importance of systematically implementing validated assessment tools adapted to the clinical environment. Given that its presence is associated with adverse outcomes such as cognitive and functional decline, increased hospital length of stay, and even institutionalization, the role of nursing staff becomes essential. Their active participation in early detection, risk factor mitigation, and comprehensive care contributes significantly to improving clinical outcomes and the quality of life of geriatric patients. Consequently, the clinical practice application of this study consists of fostering greater awareness of the utility of systematic screening in EDs, accompanied by specific training programs oriented toward the identification and management of delirium. Furthermore, it is essential to promote preventive interventions designed from a multidisciplinary perspective, as well as to establish post-discharge follow-up protocols that include a comprehensive geriatric assessment and personalized care plans for patients diagnosed with this syndrome [40,47,48].

### 4.4. Meta-Analysis

The meta-analysis encompassed a substantial total sample of 164,478 patients, yielding a pooled prevalence estimate of 20.6% (95% CI: 16.95–24.42%) for delirium in geriatric ED settings. This quantitative finding reinforces the premise that acute confusional state is a highly frequent occurrence among older adults in acute care environments.

However, these overall results must be contextualized by the significant heterogeneity observed across the primary studies, as indicated by an $I^{2}$ value of 99.5%. Several cohorts reported prevalence rates clustering between 11% and 15% [14,19,22,24], whereas other investigations, such as those by Soler-Sanchis et al. [20], reported considerably higher proportions, reaching 41%.

This marked variability is likely attributable to profound methodological differences across the studies. Primarily, the diverse array of screening tools employed—including the 4AT, bCAM, FAM-CAM, and REDEEM risk score—results in different sensitivities, specificities and diagnostic thresholds [49,50,51].

Despite this high degree of heterogeneity, Egger’s test detected no publication bias among the included literature. This absence of publication bias suggests that the pooled prevalence estimate of 20.6% remains robust and is not artificially inflated by the selective publication of positive or extreme results.

### 4.5. Limitations and Strengths

The present work has several limitations that must be considered when interpreting its results. In the first place, the lack of validation of some tools used, such as the REDEEM scale, limits both its interpretation and the applicability of its results in current clinical practice. Likewise, the study focuses exclusively on the geriatric population treated in the ED; therefore, it is likely that the findings may not be generalizable to other age groups or care settings, such as inpatient units, primary care, or nursing homes.

The meta-analysis data must be interpreted with caution due to the high degree of heterogeneity among the analyzed studies. For future research, a stratified analysis by variables (such as sex, age, use of diagnostic criteria, etc.) is proposed to decrease the $I^{2}$ index.

Despite these limitations, the study also possesses relevant strengths. One of them is that the included articles cover a broad geographical representation, providing a diversity of clinical contexts. Furthermore, the selection of exclusively quantitative studies provides a higher degree of objectivity in the analyzed results, supporting the robustness of the conclusions drawn. Future studies should be aimed at exploring preventive interventions and their real impact on the incidence and outcomes of delirium, designing specific training programs directed at nursing staff, as well as continuing to generate evidence supporting effective methods for recognizing and managing delirium adapted to the dynamic environment of the ED.

## 5. Conclusions

Delirium in older patients constitutes a frequent clinical problem with significant consequences in EDs. Around 20% of patients presenting to EDs may show clinical signs of delirium, although this information should be interpreted with caution. The available evidence suggests that delirium is common amongst older adults assessed in EDs. However, the considerable heterogeneity in the study populations, diagnostic criteria and screening methods limits the interpretation and generalisation of the pooled prevalence estimate. The key risk factors are advanced age, dementia, polypharmacy, and prolonged stays. Based on these findings, we reaffirm the importance of implementing preventive strategies focused on minimizing risks, alongside training programs directed at healthcare personnel—particularly nursing staff—fostering a comprehensive and coordinated approach that contributes to optimizing care, prognosis, and the quality of life of geriatric patients. Likewise, the need to incorporate validated detection tools into daily clinical practice is highlighted.

## Data Availability

No new data were created or analyzed in this study. Data sharing is not applicable to this article.

## Supplementary Materials

The following supporting information can be downloaded at: https://www.mdpi.com/article/10.3390/brainsci16080864/s1. Table S1: PRISMA checklist [52]. Table S2. Risk of bias appraisal of included studies using the JBI Critical Appraisal Checklist for Prevalence Studies.

## Appendix A

**Table A1:** Main characteristics of the included studies (n = 11).

Author and Year (Country)	Design	Sample	Epidemiology	Results	Level of Evidence & Grade of Recommendation
Anand et al., 2022 (UK) [23]	Multicenter retrospective cohort study	82,770 emergency hospital admissions of patients ≥ 65 years old across Lothian (Scotland) and Salford (England) hospitals	Delirium was present in 18% (Lothian) and 25% (Salford) of the patients assessed with the 4AT in the ED. Patients > 90 years old were more likely (66%) to complete the 4AT questionnaire compared to 58% of those aged 65–70 years.	A 4AT score ≥ 4 was associated with a 5.5-fold higher mortality in Lothian and a 3.4-fold higher mortality in Salford, longer hospital length of stay, and less mean time at home at one year compared to patients without delirium (4AT = 0).	3b/B
Béland et al., 2021 (CA) [14]	Multicenter prospective cohort study	612 independent/semi-independent patients ≥ 65 years old with a stay of ≥8 h in the ED of 5 hospitals in Quebec	ED delirium incidence of 11% (68 of 612 patients). Delirium was more frequent in patients with cognitive impairment (45% vs. 17%), greater frailty (68% vs. 50%), and older age (80.6 vs. 76.4 years).	Predictors of delirium were cognitive impairment (OR 3.6, *p* < 0.001), lack of mobilization in the ED (OR 3.3, *p* = 0.002), and longer ED length of stay (OR 1.02, *p* = 0.006).	2b/B
Mailhot et al., 2020 (USA) [15]	Prospective observational study	108 dyads of patients ≥ 70 years old and their family caregivers	The prevalence of delirium in the ED was 28% (30 out of 108 patients were CAM-positive) with a mean age of 80 years; half were men and the majority were white. A total of 54% of the patients had dementia, and previous episodes of confusion were more common among patients with delirium (47% vs. 6%).	The FAM-CAM, completed by family members, showed a sensitivity of 57%, a specificity of 83%, and a positive likelihood ratio of 3.4.	2b/B
Meged-Book et al., 2024 (Israel) [16]	Prospective observational study	Patients ≥ 65 years old attending the ED Phase T0: 23,724 Phase T1: 7451 Phase T2: 5394 Phase T3: 2471	T0 and T1: Delirium rate diagnosed by ED physicians < 1%. T2: Overall delirium rate of 1.6% among those evaluated by ED physicians, 1.9% when evaluators were present but not participating in detection, and 14.9% among patients evaluated with the bCAM. T3: The diagnosis rate returned to <1%. Patients with a mean age of 79.7 ± 8.1 predominated, and dementia was significantly associated with delirium.	Screening with dedicated evaluators was implemented, increasing delirium detection. Upon their withdrawal, the diagnosis rate returned to low baseline values.	2b/B
Morales-Puerto et al., 2024 (Spain) [17]	Prospective diagnostic accuracy study	380 patients ≥ 65 years old admitted to the ED without a diagnosis of dementia or severe mental disorder	Delirium prevalence according to the 4AT was 12.6%, compared to 6.3% according to the DRS-R-98.	Patients with delirium presented a higher risk of mortality and cognitive impairment, and their mean age was 83.5 years. The Spanish version of the 4AT showed a sensitivity of 95.83% and a specificity of 92.98%.	2b/B
Mueller et al., 2023 (USA) [18]	Retrospective cohort study	70,550 electronic health records extracted for patients ≥ 65 years old hospitalized from the ED between 1 January 2014 and 31 December 2020	In the studied population, 28.4% of patients hospitalized from the ED tested positive for delirium within the first 72 h of hospitalization.	The best machine learning model was the GBM with an AUC of 0.839 (95% CI: 0.837–0.841). At a 90% sensitivity threshold, the specificity was 53.5%, the positive predictive value was 43.5%, and the negative predictive value was 93.1%.	3b/B
Oliveira et al., 2022 (USA) [24]	Prospective observational study	967 patients ≥ 75 years old in the ED	The incidence of delirium in the ED was 11.1%. Hypoactive delirium accounted for 74.8% of the cases.	The REDEEM risk score was developed, based on 10 variables available at triage and early medical history. The model had an AUC of 0.901 (95% CI: 0.864–0.938). Using a threshold of ≥11, sensitivity was 84.1% and specificity was 86.6%.	2b/B
Pouw et al., 2023 (Netherlands) [19]	Prospective observational study	71 geriatric patients and 49 older patients hospitalized in the ED	The prevalence of delirium in the ED was 6.1% for older patients and 11.6% for geriatric patients using the 4AT tool, which had a sensitivity of 67% and a specificity of 83%.	The Dutch 4AT had a sensitivity in older and geriatric patients of 0.67 (95% CI: 0.09–0.99) and 0.88 (95% CI: 0.47–0.99), respectively. Specificity was 0.83 (95% CI: 0.69–0.92) in the ED population and 0.69 (95% CI: 0.56–0.80) in the acute geriatric ward.	2b/B
Soler-Sanchis et al., 2023 (Spain) [20]	Retrospective case–control study	Adults ≥ 65 years old; cases: patients diagnosed with delirium, excluding alcohol or substance abuse (n = 128); controls: randomized from the remaining patients (n = 128)	Incidence of delirium in the ED: 0.7%. The triage flowcharts most associated with delirium were unwell adult (OR = 3.04, 95% CI: 1.82–5.1) and bizarre behavior (OR = 16.06, 95% CI: 3.72–69.29).	Significant risk factors identified included dementia (OR = 3.98), previous stroke (OR = 4.79), acute neurological disease (OR = 2.39), falls in the last 30 days (OR = 2.46), and incontinence (OR = 2.46). The discriminators with the strongest association with delirium were rapid onset (OR = 3.3) and recent neurological deficit < 24 h (OR = 4.76).	3b/B
Soler-Sanchis et al., 2024 (Spain) [21]	Prospective diagnostic accuracy study	370 patients ≥ 65 years old, of which 216 were examined at the Francesc de Borja Hospital (Gandía)	Prevalence of delirium in the ED was 41.1% in the ≥65 population; high incidence in those over 80 years old, with the probability tripling in those with dementia (OR = 2.6, 95% CI: 1.58–4.26, *p* < 0.001). No significant differences between sex or priority groups.	A score of 3 points or more on the 4AT scale showed a sensitivity of 85.1% and a specificity of 66.9% for detecting delirium at triage without increasing care time.	2b/B
Vardy et al., 2020 (UK) [22]	Quasi-experimental quality improvement (QI) study using PDSA methodology	Patients ≥ 65 years old admitted to the ED of the Salford Royal NHS Foundation Trust	In 2016/2017, 1899 cases of delirium were detected in patients ≥ 65 years old (11% of non-elective admissions). Following the intervention in 2017/2018, 2546 cases (15%) were detected, with a reduction in hospital length of stay from 13 to 11 days.	The implementation of an electronic registry improved delirium detection from 3% to 43%, reducing hospital length of stay by 2 days.	2b/B

AUC: Area Under the Curve; bCAM: Brief Confusion Assessment Method; CA: Canada; CAM: Confusion Assessment Method; CI: Confidence interval; DRS-R-98: Delirium Rating Scale-Revised-98; ED: emergency department; FAM-CAM: Family Confusion Assessment Method; GBM: Gradient Boosting Classifier; OR: odds ratio; REDEEM: REcognizing DElirium in geriatric Emergency Medicine; UK: United Kingdom; USA: United States of America; 4AT: 4 “A”s Test.
