# Supplementary material for: Early Diagnosis of Delirium in Geriatric Patients in Emergency Departments: A Systematic Review and Meta-Analysis
Source: Brain Sci. 2026 Aug 15;16(8):864. doi: 10.3390/brainsci16080864 (PMC13511352; doi:10.3390/brainsci16080864)
Supplement: Supplementary file 1 [file brainsci-16-00864-s001.zip › brainsci-4478829-supplementary.pdf]

Table S1: PRISMA checklist.

Section and Topic	Item #	Checklist item	Location where item is reported
**TITLE**			
Title	1	Identify the report as a systematic review.	Line 3
**ABSTRACT**			
Abstract	2	See the PRISMA 2020 for Abstracts checklist.	Lines 45
**INTRODUCTION**			
Rationale	3	Describe the rationale for the review in the context of existing knowledge.	Lines 114-117
Objectives	4	Provide an explicit statement of the objective(s) or question(s) the review addresses.	Lines 124-126
**METHODS**			
Eligibility criteria	5	Specify the inclusion and exclusion criteria for the review and how studies were grouped for the syntheses.	Lines 144-154
Information sources	6	Specify all databases, registers, websites, organisations, reference lists and other sources searched or consulted to identify studies. Specify the date when each source was last searched or consulted.	Line 132-133
Search strategy	7	Present the full search strategies for all databases, registers and websites, including any filters and limits used.	Lines 137-142
Selection process	8	Specify the methods used to decide whether a study met the inclusion criteria of the review, including how many reviewers screened each record and each report retrieved, whether they worked independently, and if applicable, details of automation tools used in the process.	Lines 162-165
Data collection process	9	Specify the methods used to collect data from reports, including how many reviewers collected data from each report, whether they worked independently, any processes for obtaining or confirming data from study investigators, and if applicable, details of automation tools used in the process.	Lines 162-165
Data items	10a	List and define all outcomes for which data were sought. Specify whether all results that were compatible with each outcome domain in each study were sought (e.g. for all measures, time points, analyses), and if not, the methods used to decide which results to collect.	Lines 157-159
	10b	List and define all other variables for which data were sought (e.g. participant and intervention characteristics, funding sources). Describe any assumptions made about any missing or unclear information.	Lines 157-159
Study risk of bias assessment	11	Specify the methods used to assess risk of bias in the included studies, including details of the tool(s) used, how many reviewers assessed each study and whether they worked independently, and if applicable, details of automation tools used in the process.	Lines 178-184
Effect measures	12	Specify for each outcome the effect measure(s) (e.g. risk ratio, mean difference) used in the synthesis or presentation of results.	Lines 553-805
Synthesis	13a	Describe the processes used to decide which studies were eligible for each synthesis (e.g. tabulating the study intervention	Lines 157-159

Section and Topic	Item #	Checklist item	Location where item is reported
methods		characteristics and comparing against the planned groups for each synthesis (item #5)).	
	13b	Describe any methods required to prepare the data for presentation or synthesis, such as handling of missing summary statistics, or data conversions.	Lines 156-158, 178-184
	13c	Describe any methods used to tabulate or visually display results of individual studies and syntheses.	Lines 156-158, 178-184
	13d	Describe any methods used to synthesize results and provide a rationale for the choice(s). If meta-analysis was performed, describe the model(s), method(s) to identify the presence and extent of statistical heterogeneity, and software package(s) used.	Lines 173-177
	13e	Describe any methods used to explore possible causes of heterogeneity among study results (e.g. subgroup analysis, meta-regression).	Lines 173-177
	13f	Describe any sensitivity analyses conducted to assess robustness of the synthesized results.	Lines 173-177
Reporting bias assessment	14	Describe any methods used to assess risk of bias due to missing results in a synthesis (arising from reporting biases).	Lines 178-184
Certainty assessment	15	Describe any methods used to assess certainty (or confidence) in the body of evidence for an outcome.	Lines 168-170
**RESULTS**			
Study selection	16a	Describe the results of the search and selection process, from the number of records identified in the search to the number of studies included in the review, ideally using a flow diagram.	Lines 188-193
	16b	Cite studies that might appear to meet the inclusion criteria, but which were excluded, and explain why they were excluded.	Lines 532-569
Study characteristics	17	Cite each included study and present its characteristics.	Lines 569-821
Risk of bias in studies	18	Present assessments of risk of bias for each included study.	Table S2
Results of individual studies	19	For all outcomes, present, for each study: (a) summary statistics for each group (where appropriate) and (b) an effect estimate and its precision (e.g. confidence/credible interval), ideally using structured tables or plots.	Lines 569-821
Results of syntheses	20a	For each synthesis, briefly summarise the characteristics and risk of bias among contributing studies.	Table S2
	20b	Present results of all statistical syntheses conducted. If meta-analysis was done, present for each the summary estimate and its precision (e.g. confidence/credible interval) and measures of statistical heterogeneity. If comparing groups, describe the direction of the effect.	Lines 832-833
	20c	Present results of all investigations of possible causes of heterogeneity among study results.	Lines 832-833
	20d	Present results of all sensitivity analyses conducted to assess the robustness of the synthesized results.	Lines 832-833

Section and Topic	Item #	Checklist item	Location where item is reported
Reporting biases	21	Present assessments of risk of bias due to missing results (arising from reporting biases) for each synthesis assessed.	Table S2
Certainty of evidence	22	Present assessments of certainty (or confidence) in the body of evidence for each outcome assessed.	Lines 569-821
**DISCUSSION**			
Discussion	23a	Provide a general interpretation of the results in the context of other evidence.	Lines 321-331
	23b	Discuss any limitations of the evidence included in the review.	Lines 456-476
	23c	Discuss any limitations of the review processes used.	Lines 456-472
	23d	Discuss implications of the results for practice, policy, and future research.	Lines 472-476
**OTHER INFORMATION**			
Registration and protocol	24a	Provide registration information for the review, including register name and registration number, or state that the review was not registered.	Line 134-135
	24b	Indicate where the review protocol can be accessed, or state that a protocol was not prepared.	Line 134-135
	24c	Describe and explain any amendments to information provided at registration or in the protocol.	Line 134-135
Support	25	Describe sources of financial or non-financial support for the review, and the role of the funders or sponsors in the review.	Line 501
Competing interests	26	Declare any competing interests of review authors.	Line 509
Availability of data, code and other materials	27	Report which of the following are publicly available and where they can be found: template data collection forms; data extracted from included studies; data used for all analyses; analytic code; any other materials used in the review.	Lines 491-492

From: Page MJ, McKenzie JE, Bossuyt PM, Boutron I, Hoffmann TC, Mulrow CD, et al. The PRISMA 2020 statement: an updated guideline for reporting systematic reviews. BMJ 2021;372:n71. doi: 10.1136/bmj.n71

For more information, visit: http://www.prisma-statement.org/

Table S2. Risk of bias appraisal of included studies using the JBI Critical Appraisal Checklist for Prevalence Studies.

Study / Reference	Q1	Q2	Q3	Q4	Q5	Q6	Q7	Q8	Q9	Overall Risk of bias
Anand et al., 2022 [23]	Y	Y	Y	Y	Y	Y	Y	Y	Y	Low
Béland et al., 2021 [14]	Y	Y	Y	Y	Y	Y	Y	Y	Y	Low
Mailhot et al., 2020 [15]	Y	Y	N	Y	Y	Y	Y	Y	Y	Moderate
Meged-Book et al., 2024 [16]	Y	Y	Y	Y	Y	Y	Y	Y	Y	Low
Morales-Puerto et al., 2024 [17]	Y	Y	Y	Y	Y	Y	Y	Y	Y	Low
Mueller et al., 2023 [18]	Y	Y	Y	Y	Y	Y	Y	Y	Y	Low
Oliveira et al., 2022 [24]	Y	Y	Y	Y	Y	Y	Y	Y	Y	Low
Pouw et al., 2023 [19]	Y	Y	N	Y	Y	Y	Y	Y	Y	Moderate
Soler-Sanchis et al., 2023 [20]	Y	N	Y	Y	Y	N	N	Y	Y	High
Soler-Sanchis et al., 2024 [21]	Y	Y	Y	Y	Y	Y	Y	Y	Y	Low
Vardy et al., 2020 [22]	Y	Y	Y	Y	Y	Y	Y	Y	Y	Low

Y: Yes (criterion met). **N:** No (criterion not met).

Q1: Was the sample frame appropriate to address the target population? **Q2:** Were study participants sampled in an appropriate way? **Q3:** Was the sample size adequate? **Q4:** Were the study subjects and the setting described in detail? **Q5:** Was the data analysis conducted with sufficient coverage of the identified sample? **Q6:** Were valid methods used for the identification of the condition? **Q7:** Was the condition measured in a standard, reliable way for all participants? **Q8:** Was there appropriate statistical analysis? **Q9:** Was the response rate adequate, and if not, was the low response rate managed appropriately?
